# Surface‐Sensitive Characterization of Nujol Interaction with CaCO_3_ (104) Surfaces

**DOI:** 10.1002/cphc.202500766

**Published:** 2026-03-31

**Authors:** Lanna I. M. Sinimbu, Jesana M. Loreto, Maria Luiza Dorneles, Igor Coelho, Emilia Annese, Fernando Stavale

**Affiliations:** ^1^ Brazilian Center for Research in Physics (CBPF) Rio de Janeiro‐RJ Brazil; ^2^ Instituto de Física Universidade do Rio de Janeiro Rio de Janeiro Brazil

**Keywords:** adsorption, AFM, alkane, CaCO_3_ (104), IRRAS, surface reactivity, XPS

## Abstract

In this study, we investigated the adsorption of Nujol, composed mainly of n‐dodecane molecules, on pristine and hydrated CaCO_3_ (104) surfaces by atomic force microscopy (AFM), X‐ray photoelectron spectroscopy (XPS), and infrared reflection‐absorption spectroscopy (IRRAS). Our results reveal changes in the molecule–calcite interaction connected to differences in the substrate surface chemical composition upon hydration. XPS analysis suggests that the ideal pristine calcite forms a Ca^2+^ ‐deficient surface under hydration as a consequence of the dissolution process, whose traces were identified in the AFM topography images. Upon Nujol adsorption, both interface's chemical composition and topography indicate the formation of a continuous versus a discontinuous film (several patches and 3D islands) on pristine and hydrated calcite surfaces, respectively. Finally, the IRRAS spectral analysis allowed us to correlate the distinct film's topography to a specific binding geometry of the molecule. These findings provide clear evidence of the modification of the hydrocarbon molecule–calcite interaction that is essential for optimizing key parameters (surface composition, surface hydration, and oil adhesion) to improve technologies related to wettability alteration, particularly in the field of oil recovery process.

## Graphical abstract

The adsorption of model alkane molecules on pristine and hydrated calcites was investigated using surface‐sensitive techniques. Significant changes in the molecule–calcite interaction, associated with defect formation and hydrated species on the substrate, alter molecule adsorption. Our findings reveal that modified interfacial interactions are essential for improving the understanding of wettability changes in technologies used in oil recovery processes.

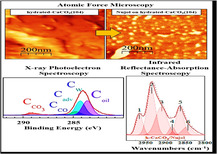

## 1 Introduction

Understanding the mechanisms governing the interaction between mineral surfaces and oil is essential for elucidating natural geochemical processes and optimizing industrial applications, such as enhanced oil recovery (EOR). The interface formation, including the molecule's adhesion energy, orientation, and coverage, is key in the petroleum recovery process since the mobility of oil species depends on the specific functional group's interaction with cationic and anionic species on the mineral surface. In this context, recent works have explored the topography and chemical composition of calcite surfaces under different conditions, revealing their dynamic reorganization [1, 2, 3].

The freshly cleaved calcite (104) surface exhibits specific structural characteristics such as atomic steps and needles oriented along defined crystallographic directions that determine its response to external agents [4, 5]. One procedure for stabilizing its surfaces consisted of a thermal conditioning that favors relaxation, reduces the structural defects, and makes its surface energy uniform [1, 6]. Another method makes use of surface hydration with deionized water (DW) to promote the release of Ca^2+^ and CO_3_^2−^ via dissolution [1]. Both localized dissolution and heterogeneous recrystallization leave specific signatures, such as pits, islands, and steps, resulting from the surface reorganization, as observed via atomic force microscopy (AFM) images [6, 7, 8], and affect the surface wettability [9].

The surface chemical composition and topography play an important role in the oil adsorption. In fact, the interaction between linear hydrocarbons and calcite was found to be dominated by van der Waals forces and to promote the formation of monolayers or multilayers depending on the crystal surface [10, 11, 12]. Nujols were predicted to preferentially align parallel to the interface formed with (104) calcite by distinct theoretical calculations, including molecular dynamics simulations and first‐principles calculations using density functional theory (DFT) [3, 13]. Despite its weak interaction with the substrate, some molecular dynamics simulations predicted that light oil molecules may form a compact layer, inhibiting the surface dissolution and altering its topographic properties [14, 15, 16]. Moreover, the oil/calcite interaction suffers a prior hydration of the single crystal that changes the interfacial chemistry, accentuating localized dissolution, ionic reorganization, and, therefore, the formation of stable organic layers and the corresponding surface wettability [4, 17].

Here, we investigated interaction between Nujol and the calcite (104) surface. Freshly cleaved calcite was subjected to a preliminary thermal treatment up to 160°C; a set of samples underwent a subsequent hydration in DW. Hereafter, the crystal will be named calcite and h‐calcite. Both calcite and h‐calcite were conditioned with Nujol [18, 19, 20]. The calcite topography varies with its surface treatment as well as oil adsorption on it. Nujol forms a dense coating on CaCO_3_ and distinct islands on h‐CaCO_3_. The film formation is strictly correlated to the preferential molecule adsorption geometry (standing or lying down). This work is expected to contribute to the understanding of the interaction between CaCO_3_ and linear hydrocarbons, which is fundamental for EOR.

## 2 Experimental Procedure

### 2.1 Materials and Sample Preparation Procedures

High‐quality commercial Nujol mineral oil (Sigma–Aldrich > 99.9% purity) was used as our model alkane molecule. Actually, attenuated total reflectance‐fourier transform infrared (ATR‐FTIR measurements were conducted on Nujol in liquid phase reveal its composed mainly by n‐dodecane molecules, as reported elsewhere [4]. The choice of Nujol was dictated by its relevance in the EOR research field, since it is one of the oil constituents and is simple enough to be modeled as a representative linear alkane, such as dodecane [4]. The pristine calcite sample were prepared by cleaving of a CaCO_3_(104) single crystals, followed by thermal treatment at 160°C for 4 h under 0.1 bar conditions in a muffle furnace. A subset of pristine samples was immersed in DW for 1 h to promote surface hydration. To address the Nujol adhesion on the calcite surfaces, each of them was immersed in Nujol for 1 h and then gently dried under a nitrogen (N_2_) stream.

All conditioned surfaces were analyzed using AFM, X‐ray photoelectron spectroscopy (XPS), and infrared reflection‐absorption spectroscopy (IRRAS). All measurements were performed on freshly prepared samples transported in an N_2_‐filled container and immediately introduced into either the AFM or XPS/IRRAS corresponding experimental setup.

### 2.2 Sample Morphological and Chemical Characterizations

A Nanoscope V MultiMode 8 AFM (Bruker) operating in an ambient‐controlled enclosure at lower than 20% relative humidity at 20–25°C. The AFM topographic images were obtained in contact mode utilizing silicon tip with a resonance frequency ranging from 90 to 240 kHz. All images were analyzed using WSxM software.

XPS and IRRAS measurements were performed in a ultra‐high vacuum (UHV) multichamber system with a base pressure of 10‐^10^ mbar. XPS measurements were performed using a monochromatic X‐ray source, FOCUS 600 NAP, and SPECS PHOIBOS‐NAP analyzer at normal emission geometry. Sample charging was compensated using an electron flood gun (I, V) = (50 μA, 3–10 V). Although different methods were used to reference the binding energy of XPS spectra either using the core or vacuum level of C–C/C–H component of the C 1s spectrum from adventitious carbon [21, 22]. Calcite is insulating sample and its work function measurement (required to evaluate specimen vacuum level) is made difficult for its surface charging. Therefore, the binding energy of 346.6 eV (Ca 2p _3/2_) was used to calibrate the *x*‐axis.

The spectral resolution was determined as 0.5 eV for high‐resolution spectra, utilizing the Ag 3d_3/2_ core level full‐width‐half‐maximum (FWHM) measured from a UHV cleaned Ag (001) single crystal reference sample (pass energy 15 eV). The XPS data analysis was carried out using the CASAXPS software [23]. The XPS quantification of survey spectra was obtained after Shirley background subtraction, accounting for the photoemission cross section and inelastic mean free path. The high‐resolution spectra were analyzed after Shirley background subtraction, and the difference spectra (raw background) are used in the figures. Each core‐level spectra, C 1s, Ca 2p, and O 1s, was fitted using a number of components with a line shape approximated by a GL function (product of Gaussian (30%) and Lorentzian (70%) line shape). The identification of C 1s core‐level components was obtained through a careful comparison of different calcite surfaces from the literature [4, 5, 10, 24, 25]. In the case of Ca 2p core level, the spectra were carefully fit, the spin–orbit components ratio was set to 0.6, and the two satellite features were located at around 9 and 12.5 eV from the main Ca 2p peak.

In addition, the molecule and substrate core‐level peak intensity changes were also conducted to verify the molecular layer thickness. In general, such evaluation depends on the formation of homogeneous thin‐film coverage [26]. In contrast, our results, discussed in the following sections, indicate that in some of the explored molecule–calcite adsorption conditions, the interface is characterized by irregular islands. Yet, we have evaluated the film coverage as a mean to semiquantitatively verify, comparatively, the molecule content adsorbed on the calcite surface. To this end, we have estimated from XPS using the relation $t=-\lambda \cos\theta \left(1+\frac{\frac{I_{0}}{S_{0}}}{\frac{I_{s}}{s_{s}}}\right)$ where, *t* is the film thickness, *λ* is the attenuation length of the cation or metal photoelectrons in the film, *θ* is the emission angle measured with respect to the surface normal, *I*_0_ and *I*_s_ are the measured peak intensities from film and substrate, respectively, and *S*_0_ and *S*_s_ are their sensitivity factors [26]. The attenuation length was obtained with Quase software for C_12_H_26_ compounds and found to be *λ* = 3.4 nm at kinetic energy of 1200 eV [27].

IRRAS measurements were performed using a Bruker VERTEX 70v infrared spectrometer and a liquid nitrogen‐cooled mercury cadmium telluride detector. The specimens were investigated by IRRAS after the XPS measurements, using non‐polarized infrared light at a grazing incidence angle of 83° along the CaCO_3_ (104) [421] crystallographic direction. IR spectra were acquired in the mid‐infrared region (4000 to 600 cm^−1^), with a spectral resolution of 4 cm^−1^ and 512 scans at room temperature. The IRRAS background spectrum was acquired using the same crystal (calcite and h‐calcite) prior to Nujol conditioning. This procedure allowed the identification of the vibrational bands associated with adsorbed molecules. All spectra are displayed in absorbance units, as well as baseline‐corrected and normalized with respect to the most intense peak. To facilitate a straightforward comparison between the adsorbed Nujol and its expected vibrational bands, pure Nujol was measured in a flow‐through attachment for ATR‐FTIR. The analyses focused on the C–H stretching bands (3000–2800 cm^−1^) of Nujol. Notably, it's well reported in the literature that polarized IR light IRRAS measurements can be employed to directly provide information about the molecule's adsorption sites and orientation on thin film and metal surfaces; however, their application on nonconductive samples such as calcite represents a significant challenge [28, 29, 30]. In fact, the characteristic calcite dielectric response does not necessarily fulfill the ideal conditions for either polarized light surface enhancement or cancellation of components. Therefore, herein we limited our investigation to exploring IRRAS spectra using non‐polarized infrared reflection, aiming to evaluate the modification of the C–H stretching bands shifts and relative intensity to changes on the molecule–molecule and molecule–substrate interaction. As discussed in the following sections, our spectral analysis is based on comparison between our own data and those reported in the literature for Nujol molecules adsorbed on metal substrates as a means to establish correlations between the molecule interaction strength and expected change on its orientation.

## 3 Results and Discussion

### 3.1 CaCO_3_ Versus h‐CaCO_3_: Topography and Surface Chemical Composition

It was established that freshly cleaved calcite (104) surfaces exposed to air are covered by at least two monolayers of bound water [31, 32].

**FIGURE 1:**
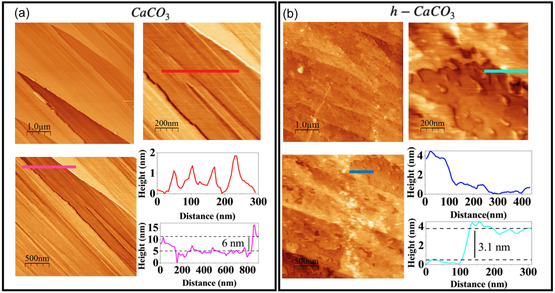
AFM topographic images of (a) CaCO_3_(104) and (b) h‐CaCO_3_(104) surfaces acquired in contact mode. To emphasize surface features at different magnifications, images of scale bars (1 µm, 500 nm, and 200 nm) are illustrated for each sample. The colored lines in the images correspond to the positions where the height profiles (same color code) were taken.

Figure 1a–b shows the surface topography of the (104) planes of CaCO_3_ and h‐CaCO_3_. The calcite surface exhibits an irregular distribution of terraces, reaching height profiles of up to 6 nm (pink curve). A closer look at surface (scale of 200 nm) shows multiple steps within a single terrace, with heights ranging from 1 to 2 nm and widths between 90 and 200 nm (red curve). These profiles are characteristic of freshly cleaved calcite (104) surfaces [26]. In Figure 1b, the h‐calcite surface displays discontinuous step edges, irregular terraces, and the appearance of nanoscale islands, with a height profile of approximately 3.1 nm (light–blue curve). These features indicate an active surface dissolution process, generally associated with ionic redistribution and heterogeneous recrystallization occurring at the mineral–water interface [33, 34, 35]. The AFM topographic profile (dark–blue curve) reveals nonhomogeneous terraces, with heights around 4 nm. Such variations indicate a significant reduction in structural order and an increase in surface roughness, pointing to a higher degree of solvation on the h‐calcite surface.

**FIGURE 2:**
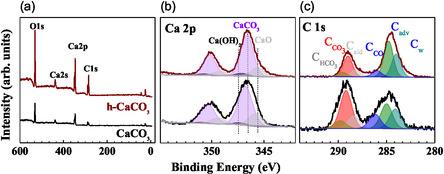
XPS survey (a), Ca 2p (b), and C 1s (c) core‐level spectra of calcite (black line) and h‐calcite (wine line). The deconvolution of each spectrum is highlighted with its respective components. For the sake of clarity, the high‐resolution spectra were normalized to the most intense feature.

Figure 2 displays survey XPS spectra of calcite (black) and h‐calcite (wine), and the most intense electron transitions for each element (O, Ca, and C) are highlighted. No impurities were revealed in the survey spectra. The relative intensity redistribution in XPS survey (Figure 2a) and high‐resolution core‐level spectra (Ca 2p and C 1s, Figure 2b,c) of calcite and h‐calcite reflects their distinct surface chemical composition, as quantified through the normalized area of XPS peaks (Table 1S in Supporting Information).

All high‐resolution spectra of calcite surfaces exhibit broader and less defined features. The Ca 2p spectra consist of spin–orbit doublets (Ca 2p_1_/_2_ – Ca 2p_3_/_2_) separated by approximately 3.6 eV (Figure 2b). The main Ca 2p_3_/_2_ peak appears at 346.6 eV and second and third components at 345.6 eV and 347.2 eV, respectively: Each of them indicates a different chemical environment for Ca. Figure 2c shows the C 1s core‐level spectra, composed of a main carbonate peak at 290 eV (C_CO3_), a hydrated carbonate component at 290.7 eV (C_HCO3_), and signals at lower binding energies attributed to R–COO aldehydes, adventitious carbon (C_adv_, 284.7 eV), carbon in CO groups (285.7 eV), and water‐related carbon (C_w_, 284 eV). In previous works [5, 10, 25], we studied the behavior of freshly cleaved calcite when hydrated in controlled condition (partial pressure of H_2_O of around 2 mbar) and by immersion in DW. In both cases, we observed the evolution of C 1s core‐level spectra and identified the asymmetric shape of C adventitious feature that could fit by addition component indicated as C_w_. This component was found to increase by raising the water pressure content in UHV chamber. In the current work, the evolution of C_w_ can be used to assess the effect of hydration process on the surface.

The h‐calcite shows a decrease in the Ca/C_CO33_ ratio from 0.9 to 0.8 compared to the calcite sample, indicating partial leaching of Ca^2+^ ions from the surface. This supports the hypothesis of surface dissolution and the formation of vacancy sites upon exposure to water. The corresponding increase in the C_w_ component (from 19% to 37%) is consistent with the formation of a water layer beside surface hydroxylation [25], as previously reported [6, 36].

### 3.2 Nujol Interaction with CaCO_3_ and h‐CaCO_3_

**FIGURE 3:**
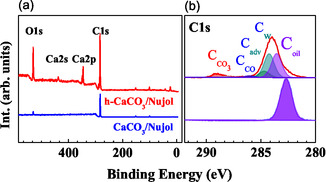
XPS survey (a) and high‐resolution C1s (b) spectra of calcite/Nujol (blue) and h‐calcite/Nujol (red). In (b), all C1s peak components are identified.

As discussed above, the pristine and hydrated calcite (104) surfaces are expected to present distinct mechanisms of interaction with the Nujol due to changes on their atomic relative concentration and morphology. The amount of Nujol adsorbed on the surface can be evaluated by the XPS spectra. Figure 3 displays the survey XPS and C 1s core‐level spectra of calcite/Nujol and h‐calcite/Nujol. On the one hand, the XPS spectra of the calcite/Nujol specimens showed mainly C 1s feature (i.e., as well as a rather weak O 1s peak), indicating that the molecule film mostly covers the surface and therefore strongly attenuate the expected photoemission peaks originating from the substrate, namely Ca and CO_3_. On the other hand, the h‐calcite/Nujol interface presents both Nujol and calcite spectral features with a Nujol content quantified as roughly 45% of the overall C 1s core‐level spectra. In this case, the molecule adsorption does not significantly attenuate the substrate electron emission. The appearance of the XPS spectra in the two cases can be attributed to the formation of a film with distinct packing on the surface. The possible scenarios may include the formation of continuous films versus patches, and the arrangement of Nujol molecules in either a flat or upright configuration, or even a mixture of both geometries. Further pieces of information about the orientation of the molecules and their interaction with the substrate can be obtained by IRRAS measurements and comparative analysis of both specimens.

**FIGURE 4:**
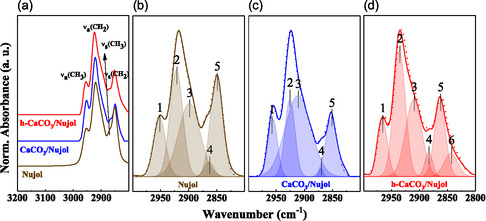
(a) IRRAS spectra corresponding to the vibrational region from 3000 to 2800 cm^−1^ of pure Nujol (brown), Nujol adsorbed on calcite/Nujol (navy blue), and h‐calcite/Nujol (red). In panel (a), each peak is labeled according to the involved CH_2_ and CH_3_ vibration bands (symmetric and asymmetric and resonant) within the Nujol molecule. Panels (b,c,d) show spectral deconvolutions of pure Nujol, calcite/Nujol, and h‐calcite/Nujol, respectively. In panels (b–d), each spectral component is identified as a number as follows: 1 ‐ ν_ass_CH_3_ at 2954 cm^−1^, 2 ‐ ν_ass_CH_2_ at 2954 cm^−1^, 3 ‐ ν_F_ at 2900 cm^−1^, 4 ‐ ν_sym_ CH_3_ at 2872 cm^−1^, and 5 ‐ ν_sym_ CH_2_ at 2852 cm^−1^_._ .

To this end, Figure 4a panel shows the IRRAS spectra obtained for pure (liquid phase) Nujol (brown), Nujol adsorbed on calcite (navy), and on h‐calcite (red) in the range 3000–2800 cm^−1^. The IRRAS spectra of Nujol adsorbed on calcite reproduce the main spectral features observed in the liquid Nujol reference spectra [4]. However, a few new additional features and relative intensity modifications may be used to infer its interaction with the substrate. For instance, a noticeable intensity reduction of the C–H stretching bands (~2924 and 2854 cm^−1^) was observed in both samples. The calcite/Nujol sample exhibits lower intensity than the h‐calcite/Nujol, suggesting a greater affinity of the oil with the hydrated surface, likely favored by the appearance of new adsorption sites due to surface dissolution induced by DW hydration [25].

A more detailed analysis of the vibrational and conformational changes induced by adsorption was obtained through its spectral band deconvolution. The Nujol (Figure 4b) and calcite/Nujol (Figure 4c) band profiles were fitted by five components identified as vibration modes (ν_ass_ CH_3_ at 2954 cm^−1^, ν_ass_ CH_2_ at 2923 cm^−1^, ν_sym_ CH_3_ at 2872 cm^−1^, and ν_sym_ CH_2_ at 2852 cm^−1^). The CH_2_ asymmetric stretching band (ν_ass_ CH_2_), assigned at 2923 cm^−1^, has a contribution from a hidden component at 2900 cm^−1^, identified as a Fermi resonance, ν_F_ [37], usually present in alkyl chains with at least two nearly equivalent neighboring CH_2_ groups [38]. The deconvolution of h‐calcite/Nujol (Figure 4d) shows an additional component at ~ 2840 cm^−1^, identified as ν_a_ (CH_2_) [39]. Details about the curve fitting (peak center in cm^−1^, FWHM, and number of components) for each specimen can be found in Table 1S in Supporting Information. It is worth noticing that the FWHM of CH_3_ groups decreases in calcite/Nujol with respect to the spectrum of pure Nujol. This reduction indicates that these Nujol groups are more rigidly organized due to the interaction with the underlying surface; in fact, this parameter describes the conformational disorder within the molecule film organization [4, 25]. Bands 2 and 4 are associated with CH_3_ asymmetric and CH_2_ symmetric vibration bands, respectively, and showed a significant decrease in their area upon interaction with the substrate. This alteration may suggest that part of the molecules is confined at the interface.

Such spectral behavior is consistent with findings from *in situ* vibrational spectroscopy studies, where the adsorption of the molecule can induce structural rearrangements and alter vibrational intensity due to changes in molecular orientation and interaction with active sites [40]. These effects may involve not only suppression of specific modes but also the emergence of new modes resulting from stronger chemical interactions (e.g., through hydrogen bonding or coordination to surface atoms). Thus, the observed reduction in band area does not necessarily indicate the absence of CH_3_ or CH_2_ groups but rather reflects modifications in their vibrational response due to adsorption‐induced conformational and orientation changes.

Furthermore, band 3 (ν_F_) presents a positive wave number shift (+11 cm^−1^). This band arises from molecule–molecule interaction, and its modification may be associated with a new vibrational coupling regime viable when the Nujol self‐organization at the surface enhances this interaction. According to XPS results Figure 3a), the amount of Nujol is such that it covers the whole substrate, attenuating significantly the underlying substrate signal. This is compatible with an enhanced molecule–molecule interaction when the Nujol is packed with a higher density, and therefore parallel to the substrate surface normal. This is in agreement with previous results where a thicker Nujol film corresponding to a dense molecule organization was already observed for longer‐chain hydrocarbon molecules such as C_32_H_66_ adsorbed on Si [41].

On the other hand, the h‐calcite/Nujol sample shows distinct spectral changes: a reduction of areas of the vibrational bands 1 (ν_ass_CH_3_) and band 3 (ν_F_). The first change can be interpreted as the involvement of the CH_3_ functional group in the interaction with the surface, whereas the intensity reduction by 16.3% of band 3 indicates a weaker interaction between molecule–molecule. Both spectral modifications are consistent with a stronger interaction of the Nujol with the h‐calcite surface. Bands 2 and 5 (CH_2_) increase in area (+8% and + 1.3%, respectively), suggesting greater conformational freedom and reduced interfacial restriction [42]. Additionally, a new band appeared at ~ 2840 cm^−1^ (band 6) [39], and it is attributed to a possible structural reorganization of the aliphatic chains on the hydrated surface. This behavior may be related to the generation of surface vacancies caused by the dissolution of calcite, creating preferential adsorption sites for CH_2_ segments [25]. The shifts observed were more uniform, with all the bands moving to higher wave numbers: band 1 shifted by + 6 cm^−1^, band 2 by + 7 cm^−1^, band 3 by + 10 cm^−1^, and bands 4 and 5 by + 5 cm^−1^.

**FIGURE 5:**
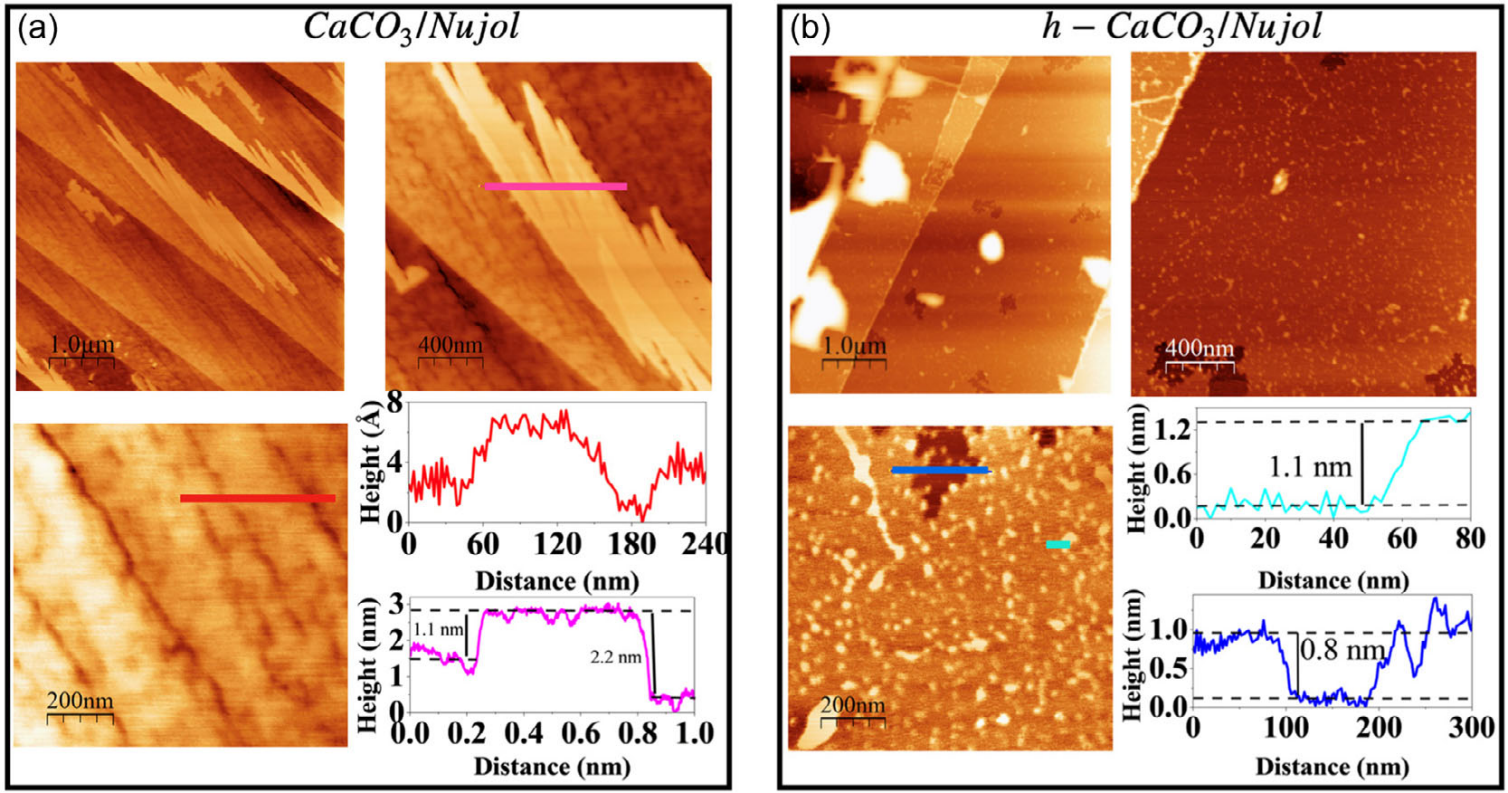
AFM images of the (a) CaCO_3_/Nujol and (b) h‐CaCO_3_/Nujol interfaces. In (a), the CaCO_3_/Nujol interface exhibits film covering well‐defined terraces in images with scale bar of 1 µm 400 nm, and 200 nm and their corresponding topographic profiles. In (b), the h‐CaCO_3_/Nujol interface shows discontinuous steps, irregular terraces, and nanoscale islands; images at 1 µm (upper left), 400 nm (upper right), and 200 nm (lower left) are displayed along with their respective profiles. Colored lines indicate in the images corresponds to the position where the height profiles were extracted.

The topographic analysis of the Nujol adsorbed on each substrate reveals distinct behaviors between the non‐hydrated and hydrated surfaces, i.e., CaCO_3_/Nujol and h‐CaCO_3_/Nujol, as shown in Figure 5a,b. The AFM image of the CaCO_3_/Nujol sample shows a surface with homogeneous coverage, adapting to the irregular morphology of the substrate and evidencing the formation of a continuous organic film. In Figure 5a, the topographic profile (pink curve) shows height variations of ≈1.1 nm and 2.2 nm, corresponding to the deposition of the film over the calcite needles, while the red curve indicates slight fluctuations on the angstrom scale, reflecting subtle local irregularities of the film. Considering that the length of an Nujol molecule is about 1.7 nm when fully oriented vertically, the observed values suggest adsorption at an inclined angle relative to the surface, resulting in a partially organized film structure [5, 25].

In contrast, Figure 5b shows the topographic images of the h‐calcite/Nujol sample, in which a heterogeneous morphology is observed, predominantly composed of islands with heights around 1.1 nm (as shown by the light–blue curve) and a discontinuous film. The topographic profile reveals more pronounced height variations, with steps of approximately 0.8 nm (as shown by the dark–blue curve), as well as microstructures indicating a disordered accumulation and possible co‐adsorption of oil and reorganized ionic residues originating from the hydration stage. These modifications highlight the role of prior hydration in reorganizing the Nujol–calcite interface, thereby influencing the subsequent adsorption of molecules and the formation of additional layers. Such observations are consistent with studies demonstrating the formation of multiple layers at the calcite/oil interface, which directly affect wettability, surface electronic structure, and the stability of the adsorbed layers [12, 14, 16, 17, 25].

## 4 Mechanism of Nujol Adsorption and Adhesion to CaCO_3_ Surfaces

The adsorption of Nujol onto carbonate mineral surfaces is governed by interfacial physicochemical interactions that depend on surface composition, crystallographic structure, and hydration state. Freshly cleaved calcite (calcite) provides a chemically uniform surface dominated by Ca^2+^ ions, which promote van der Waals interactions with Nujol, leading to the formation of thin, homogeneous films. This behavior has been discussed also in previous work based on AFM and FTIR analyses [25, 43]. Furthermore, residual hydroxyl groups and surface‐bound water molecules may vary the interaction of hydrocarbons via weak dipolar interactions with methylene groups [44]. However, upon its thermal treatment, a partial dehydration of the surface may increase the surface energy of Ca^2+^‐rich sites, slightly enhancing Nujol adhesion. The hydration following thermal treatment (h‐CaCO_3_) induces the formation of dynamic hydration layers that reduce adhesion energy, weakening the Nujol interaction.

**FIGURE 6:**
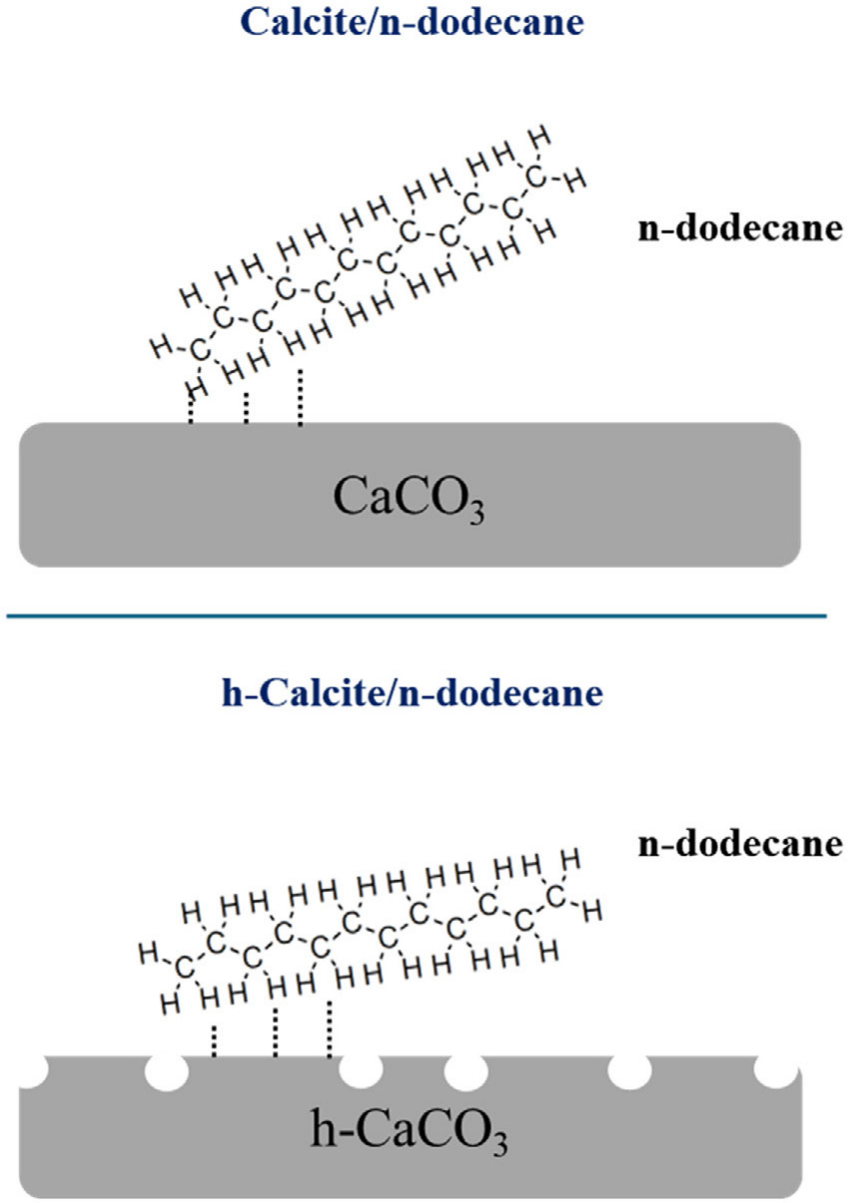
Simple schematic representation of possible Nujol (represented by a dodecane) adsorption scenarios on calcite and h‐calcite surfaces. While calcium and oxygen vacancies are indicated by white circles.

The interpretation of different adsorption on calcite and h‐calcite is based on the combined evidence from AFM, XPS, and IRRAS. AFM revealed distinct film morphologies, XPS indicated differences in surface composition, and IRRAS provided spectroscopic signatures with modified molecule–calcite interactions. While such data do not give direct atomic‐scale proof of specific defect structures, their convergence strongly supports the hypothesis that hydration promotes new anchoring sites for Nujol adsorption. On h‐calcite, the ions rearrangement and hydration layers favor molecule adsorption at the new anchoring defect sites; schematically illustrated in Figure 6.

According to IRRAS data analysis, Nujol molecules, here modeled as dodecane, formed an ordered layer in an oblique/vertical orientation relative to the (104) surface plane concomitant to an enhancement of molecule–molecule interaction, as compared to its pure liquid counterpart. Thus, a dense layer covers the underlying substrate (Figures 2 and 4a). This is in agreement with XPS results, where the disappearance of substrate features in spectra demonstrates a Nujol‐wet CaCO3 surface with an organic layer of around 19(0.5) nm thick. This is in contrast to theoretical results that predicted a lying‐down geometry for n‐dodecane on CaCO_3_. We have discussed these calculations and reports since they consider ideal and fully stoichiometric calcite surfaces and therefore do not account for the presence of steps and defects [3, 13]. In the h‐calcite/Nujol, the Ca^2+^ and CO_3_^2−^ vacancies (arising from surface dissolution) or hydroxylated group (formed with water superficial layer) turn themselves into new activated sites of the surface, which ultimately interact with methylene groups (CH2), modifying the molecule arrangement and its relative orientation. This latter is expected to play a crucial role in altering the interactions promoting the formation of the less packed and discontinuous adsorbed Nujol films (Figures 2 and 4b.). From XPS data analysis, we estimated a Nujol layer thickness of 7(0.5) nm. Although the thickness estimation is limited by the model's hypothesis, it enables us to further demonstrate the varying Nujol content on the two surfaces.

## 5 Conclusions

We have investigated the adsorption of Nujol molecules, composed mainly of n‐dodecane, on CaCO_3_ and h‐CaCO_3_ (104) surfaces. By combining AFM, XPS, and IRRAS analyses, we addressed the correlation between the surface hydration state, the chemical composition, and the adsorption mode of the hydrocarbon. In h‐calcite, the hydration promotes partial dissolution, Ca^2+^ vacancies. Nujol adhesion depends on surface termination, a dense and continuous Nujol film was favored on CaCO_3_, while a discontinuous, island like film distribution was present on h‐CaCO_3_. On CaCO_3_, the strong attenuation of hydrocarbon peak (C_oil_) in XPS spectra corresponds to thick and compact organic layer on pristine calcite. On the same substrate, IRRAS spectrum revealed narrower CH_2_ stretching bands and frequency shifts, consistent with enhanced of Nujol–Nujol interaction. On h‐CaCO_3_ surface, the discontinuous film indicates a lower Nujol uptake and their molecular main axes closer to the substrate surface as inferred from IRRAS data analysis. The mechanisms behind the adsorption of Nujol on calcite surfaces and their hydrated counterparts were proposed, and these findings may have impact on the optimization of EOR process.

## Author Contributions

**Lanna I. M. Sinimbu:** data curation (equal), formal analysis (equal), writing – original draft (equal), writing – review & editing (equal). **Jesana M. Loreto:** data curation (equal), formal analysis (equal), writing – review & editing (equal). **Maria** **Luiza Dorneles**: data curation (equal), investigation (equal), writing – review & editing (equal). **Igor Coelho**: data curation (equal), formal analysis (equal), writing – review & editing (equal). **Emilia Annese**: funding acquisition (lead), project administration (lead), supervision (lead), writing – review & editing (lead). **Fernando Stavale:** funding acquisition (lead), project administration (lead), supervision (lead), writing – review & editing (lead).

## Supporting Information

Additional supporting information can be found online in the Supporting Information section. **Supporting Table S1:** Summary of vibrational parameters obtained from spectral deconvolution of IRRAS data in the 3000–2800 cm^−1^ region for pure Nujol, Nujol adsorbed on calcite (CaCO_3_/Nujol), and Nujol adsorbed on hydrated calcite (h‐CaCO_3_/Nujol). The table reports the center position (cm^−1^), full width at half maximum (FWHM), and relative area (%) of each resolved band. Band assignments correspond to asymmetric (ν_a_) and symmetric (ν_s_) C–H stretching vibrations of methyl (CH_3_) and methylene (CH_2_) groups, including the Fermi resonance band of CH_2_.

## Funding

This study was supported by CENPES/PETROBRAS.

## Conflicts of Interest

The authors declare no conflicts of interest.

## Supporting information

Supplementary Material

## Data Availability

The data that support the findings of this study are available from the corresponding author upon reasonable request.
