# Supplementary material for: Surface‐Sensitive Characterization of Nujol Interaction with CaCO3 (104) Surfaces
Source: Chemphyschem. 2026 Mar 31;27(6):e202500766. doi: 10.1002/cphc.202500766 (PMC13037355; doi:10.1002/cphc.202500766)
Supplement: Supplementary file 1 — Supplementary Material [file CPHC-27-e202500766-s001.pdf]

Surface sensitive characterization of Nujol interaction with $CaCO_3$ (104) surfaces

Lanna I. M. Sinimbu[1,*], Jesana M. Loreto[1,*] Maria Luiza Dorneles[1], Igor Coelho[1], Emilia Annese[2,§], Fernado Stavale[1,§]

[1] Brazilian Center for Research in Physics (CBPF), Rio de Janeiro - RJ- Brazil. 22290-180

[2]Instituto de Física, Universidade do Rio de Janeiro, R. São Francisco Xavier, 524, 20950-000, Rio de Janeiro, Brazil

* authors with equal contribution

§ corresponding authors

1. Deconvolution Parameters of FTIR Spectra for alkene Adsorbed on Calcite Surfaces

Table 1S. Summary of vibrational parameters obtained from spectral deconvolution of IRRAS data in the 3000–2800 cm^{-1} region for pure Nujol, Nujol adsorbed on calcite ($CaCO_3$/Nujol), and Nujol adsorbed on hydrated calcite (h-$CaCO_3$/Nujol). The table reports the center position (cm^{-1}), full width at half maximum (FWHM), and relative area (%) of each resolved band. Band assignments correspond to asymmetric (ν_a) and symmetric (ν_s) C–H stretching vibrations of methyl (CH_3) and methylene (CH_2) groups, including the Fermi resonance band of CH_2.

**Nujol**				
**Band**	**Vibration**	**Center**	**FWHM**	**% Area**
1	ν_a (CH_3)	2955	22.6	12.2
2	ν_a (CH_2)	2923	27.0	24.2
3	Fermi(CH_2)	2900	45.0	43.4
4	νs (CH_3)	2872	19.6	3.1
5	ν_s (CH_2)	2852	23.5	17.0
**$CaCO_3$/Nujol**				
1	ν_a (CH_3)	2956	19.2	13.0
2	ν_a (CH_2)	2925	21.0	18.8
3	Fermi(CH_2)	2911	54.0	50.4

4	νs (CH_3)	2870	8.3	0.8
5	ν_s (CH_2)	2851	25.4	16.8
**h-$CaCO_3$/Nujol**				
1	ν_a (CH_3)	2961	20.7	11.7
2	ν_a (CH_2)	2930	25.0	32.2
3	Fermi(CH_2)	2910	34.1	26.8
4	νs (CH_3)	2877	16.4	4.2
5	ν_s (CH_2)	2857	23.3	18.3
6	ν_a (CH_2)	2840	28.92	6.6

Table S1 presents the parameters obtained from the deconvolution of the IRRAS spectra in the region of 3000 to 2800 cm^{-1} for liquid Nujol and adsorbed on calcite ($CaCO_3$/Nujol) and on previously hydrated calcite (h-CaCO3/Nujol). The data reveal important changes in the central positions, width at half height (FWHM) and relative areas of the vibrational bands attributed to the asymmetric (νa) and symmetric (νs) stretching modes of the methyl (CH_3) and methylene (CH_2) groups, in addition to the Fermi resonance band of CH_2. In relation to liquid Nujol, the $CaCO_3$/Nujol sample presents a positive shift of the Fermi band (+11 cm^{-1}), an increase in the FWHM (from 45.0 to 54.0 cm^{-1}) and a larger relative area (50.4%), indicating an increase in conformational rigidity and more intense vibrational coupling, associated with the interaction with the mineral surface. In the h-$CaCO_3$/Nujol sample, the introduction of a new band at 2840 cm^{-1} is observed, attributed to a structural reorganization of the CH_2 groups on the hydrated surface. The increase in the relative area of band 2 (νa CH_2) to 32.2% and of band 5 (νs CH_2) to 18.3% suggests a greater mobility of the central segments of the aliphatic chains, possibly associated with the reorganization of the adsorbed film in a conformation more parallel to the surface.
